# A Hydrogel Scaffold Incorporating Fennel Seed Extract Induces Osteogenic Differentiation in Mesenchymal Stem Cells

**DOI:** 10.1002/vms3.70460

**Published:** 2025-07-10

**Authors:** Kosar Heidari, Tayebeh Mohammadi, Leila Soltani, Mehrdad PooyanMehr

**Affiliations:** ^1^ Basic Sciences and Pathobiology Department Faculty of Veterinary Medicine Razi University Kermanshah Iran; ^2^ Department of Animal Sciences College of Agriculture and Natural Resources Razi University Kermanshah Iran

**Keywords:** BM‐MSCs, chitosan, fennel, hydrogel, osteoblast, tissue engineering

## Abstract

**Background:**

Tissue engineering utilizing hydrogels and scaffolds composed of organic and synthetic materials has emerged as a promising approach for bone repair. Chitosan, a biocompatible natural polymer, supports bone healing and possesses antibacterial and antioxidant properties. Fennel (*Foeniculum vulgare*), known for its traditional use in treating osteoporosis in females, exhibits phytoestrogenic properties.

**Objectives:**

This study investigated the osteogenic differentiation of mesenchymal stem cells (MSCs) cultured within chitosan hydrogels incorporating fennel seed extract.

**Methods:**

Fourier Transform Infrared Spectroscopy (FTIR) and Scanning Electron Microscopy (SEM) were employed to characterize the chitosan films containing fennel extract. MSCs were isolated from ovine foetal bone marrow and subjected to various concentrations of fennel hydroalcoholic extract and hydrogels containing these extracts. Cell viability was assessed using the MTT assay. Following osteoblast differentiation, gene expression of osteogenic markers (ALP, Runx2 and COL1A1) was evaluated using real‐time PCR, alongside assessments of calcium deposition, alkaline phosphatase activity and ALP expression. Alizarin red staining was performed to quantify mineralized matrix deposition.

**Results:**

The MTT assay revealed that the 0.5% extract treatment group significantly enhanced MSC proliferation. Notably, MSCs cultured on the chitosan film containing 1% fennel extract demonstrated increased numbers of hydroxyapatite deposits, elevated ALP activity and calcium content and upregulated expression of osteogenic genes (ALP, Runx2 and COL1A1). Acridine orange/ethidium bromide staining confirmed the non‐toxicity of the extract in all treatment groups.

**Conclusion:**

These findings demonstrate that chitosan hydrogels incorporating fennel seed extract can effectively induce osteogenic differentiation of MSCs, highlighting their potential as a promising strategy for bone tissue regeneration.

## Graphical abstract

Graphical description of the osteogenic differentiation of ovine foetal bone marrow mesenchymal stem cells cultured within chitosan hydrogels incorporating fennel seed extract. After 21 days, their differentiation was evaluated using morphological, biochemical and molecular assays. The results demonstrated their effective osteogenic differentiation.

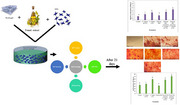

## 1 Introduction

Bone possesses a remarkable capacity for self‐repair. When a fracture is stable and anatomically well‐aligned, healing typically occurs. However, even under optimal mechanical and biological conditions, a significant proportion of fractures (approximately 10%) fail to heal completely. This highlights the need for continued research to enhance our understanding of the intricate processes involved in bone healing (Khatkar and See 2021). In regenerative medicine, bone marrow mesenchymal stem cells (BM‐MSCs) exhibit numerous advantageous qualities, including multipotency, anti‐inflammatory properties and immuno‐modulatory capabilities. This cell population possesses the capacity to promote angiogenesis and contributes to hematopoiesis. Furthermore, BM‐MSCs secrete paracrine factors that positively influence the local microenvironment and facilitate organ repair (Pittenger et al. 2019). While it is recognized that BM‐MSCs may exhibit limited engraftment potential following transplantation, they exert a significant therapeutic effect by secreting paracrine factors that modulate the local tissue microenvironment (Saeedi et al. 2021). These cells are now a desirable technique in regenerative medicine for bone regeneration due to their capacity to induce in‐vivo bone growth (Anderson 2024). By replacing damaged cells, cell therapies aim to restore the normal physiological function of the affected tissue or organ (Ajmal et al. 2023). Bone abnormalities and deformities can be addressed through tissue engineering approaches. These approaches encompass bone tissue transplantation, the design and fabrication of suitable bone scaffolds, and the implantation of cells with the capacity for proliferation and differentiation (Zhu et al. 2023). In vitro, studies have consistently shown that osteoblastic cells exhibit superior functional performance, including enhanced differentiation and matrix synthesis, when cultured within a three‐dimensional (3D) environment compared to conventional two‐dimensional (2D) monolayer systems. 3D scaffolds provide a more physiologically relevant environment by offering a larger and more complex surface area for cell adhesion, promoting cell‐cell and cell‐matrix interactions and more closely mimicking the in‐vivo microenvironment (Manzini et al. 2021; Koushik et al. 2023; Zhu et al. 2023). For the effective fabrication of tissue constructs tailored to specific therapeutic applications, mesenchymal stromal cells (MSCs) can be cultured within a biocompatible polymeric matrix (Amani et al. 2009). Hydrogels and scaffolds, fabricated from organic and synthetic materials and biopolymers, are utilized in bone restoration (Filippi et al. 2020).

A diverse array of naturally derived materials, including proteins like collagen, gelatin, laminin, keratin, elastin, fibroin, fibrin and heparin, as well as polysaccharides such as hyaluronan, chitosan and alginate, hold significant promise in addressing challenges in bone regeneration. Notably, certain natural polysaccharides, such as cellulose and dextran, exhibit inherent antimicrobial activity. These natural polymers present a compelling avenue for the development of synthetic scaffolds, potentially incorporating bioactive agents to enhance cellular differentiation, promote osteogenesis and mitigate bacterial colonization. Chitosan, specifically, is a biocompatible, naturally occurring cationic polymer recognized for its notable biological activities, encompassing both antioxidant and antibacterial properties (Lu et al. 2018; L. Li et al. 2014; Ngo and Kim. 2014; Karagozlu and Kim 2014). Chitosan, however, is insoluble in water. It is advised to use techniques e.g. carboxymethylation to address the issue of insolubility in water (L. Li et al. 2014). For instance, carboxymethyl chitosan has strong water solubility and is biocompatible (Müller et al. 2016). Driven by concerns regarding the adverse effects associated with synthetic pharmaceuticals, a significant number of researchers have directed their attention toward the therapeutic potential of medicinal plants. The *Foeniculum vulgar* (Mill plant) produces fruits and roots that are used to make an infusion that has sedative, estrogenic, analgesic and anti‐inflammatory properties. In herbal medicine, fennel finds application in the management of gastrointestinal and respiratory ailments, and it is also employed as a galactagogue to enhance lactation in nursing mothers. It has been demonstrated that fennel seeds encourage menstruation, ease labour, and lessen dysmenorrhea symptoms. Female behaviours and libido are both enhanced by fennel seed extract. Fennel essential oil has reportedly been shown to have antifungal properties. In general, fennel seed extract has been demonstrated to have estrogenic, antioxidant and anti‐hairing effects (Rather et al. 2016). Little research has examined the impact of hydrogels containing plant extracts with estrogenic chemicals on the development of MSCs into osteoblasts. In this investigation, therefore, ovine foetal bone marrow MSCs' osteoblast differentiations were assessed on hydrogel‐containing fennel extract.

## 2 Materials and Methods

### 2.1 Materials

All materials used in this study were purchased from Sigma‐Aldrich, except for those sourced from other companies, which are mentioned in the text.

#### 2.1.1 Preparation of Herbal Extract

Fennel seeds were procured from a local herbalist/a reputable supplier of medicinal plants. Crushed seeds (25 g) were macerated with 200 mL of 70% ethanol for 72 h at room temperature. The mixture was then filtered through Whatman filter paper. The filtrate was evaporated to dryness under reduced pressure in a rotary evaporator at 40°C. The resulting extract was stored at 4°C until further use.

#### 2.1.2 Identification of Extract Compounds

Gas Chromatography/Mass Spectrometry (GC‐MS) analysis was employed to identify the compounds present in the extract.

#### 2.1.3 Preparation of the Chitosan Film

Chitosan (1.5 g) was dissolved in 100 mL of deionized water containing 0.7% acetic acid. Glycerol (100 mg) was then added to the solution. The mixture was stirred vigorously for 30 min at 40°C using a magnetic stirrer to ensure complete dissolution. The resulting chitosan solution was filtered through Whatman filter paper to remove any insoluble impurities. Fennel extract was then incorporated into the chitosan solution at concentrations of 0.5% and 1% (v/v). The mixtures were homogenized using a high‐speed blender (9000 rpm) for 5 min to ensure uniform dispersion of the extract. Subsequently, 25 mL of each solution was carefully cast onto 15 cm diameter polystyrene Petri dishes. The films were allowed to dry at 25°C for 72 h under ambient conditions. Finally, the dried films were carefully peeled off the dishes and stored in sealed zip‐lock bags for further use.

#### 2.1.4 Identification of Prepared Scaffolds

Scanning electron microscopy (SEM) was employed to characterize the surface morphology of the scaffolds. Prior to imaging, the scaffold samples were gold‐coated and examined under vacuum conditions

#### 2.1.5 FT‐IR Test

FTIR analysis, utilizing transmittance mode, was conducted on 1–2 mm thick films of chitosan and chitosan‐fennel hydrogels to identify the characteristic chemical groups present. Spectra were recorded between 4000 and 650 cm⁻¹ with a resolution of 2 cm⁻¹ over 64 scans using a Paragon 1000 (Perkin‐Elmer, USA) spectrometer. The resulting FTIR spectra were normalized, and prominent vibration bands were assigned to corresponding chemical functionalities.

#### 2.1.6 Isolation and Cultivation of Bone‐Marrow MSCs

In this study, MSCs were isolated from the femur and tibia bones of an ovine foetus obtained from a local abattoir. The pregnant ewe was identified at the abattoir, and the uterus containing the foetus was immediately transported to the laboratory on ice. After cleaning the foetus with 70% ethanol, the surrounding tissues were dissected away from the bones using a sterile surgical blade. The bones were then thoroughly washed multiple times with DPBS containing antibiotics to remove any residual tissue or debris. Bone marrow aspirates were added to 5 mL of Dulbecco's Modified Eagle Medium (DMEM; Sigma, USA) supplemented with 10% Fetal Bovine Serum (FBS, Sigma, USA), 100 units/mL penicillin and 100 units/mL streptomycin (Sigma, USA). The mixture was then centrifuged at 300 × *g* for 5 min. The cell pellet was resuspended in 1 mL of Dulbecco's Modified Eagle Medium (DMEM) and seeded at a density of 5 × 10^6^ cells/cm^2^ in 75 cm^2^ culture flasks. The cultures were maintained in a humidified incubator with 5% CO_2_ at 37°C. The culture medium was refreshed every 2–3 days until the cultures reached confluence. At confluence, cells were trypsinized and sub‐cultured at a density of 5 × 10^5^ cells/cm^2^. This sub‐culturing process was repeated as needed to obtain sufficient cell numbers for subsequent experiments (Soltani et al. 2016).

#### 2.1.7 Morphology of MScs

MSCs can be identified by their characteristic morphology, including adherence to plastic surfaces, spindle‐shaped morphology, triangular cell bodies, large, euchromatic nuclei and a high nucleus‐to‐cytoplasm ratio.

#### 2.1.8 Differentiation of MSCs Into Osteogenic and Adipogenic Cells

At this stage, the capacity of passage 3 (P3) cells to differentiate into osteoblasts and adipocytes was assessed. Cells were seeded in 6‐well plates and cultured in specific induction media for each lineage. For osteogenic induction, the culture medium was replaced with osteogenic differentiation medium containing DMEM supplemented with 10% FBS, 7–10 nM dexamethasone, 10 mM β‐glycerophosphate and 50 µg/mL ascorbic acid 2‐phosphate (all from Sigma). After 21 days of incubation, mineralized matrix deposition was assessed by Alizarin Red S staining (Sigma). For adipogenic induction, the culture medium was replaced with an adipogenic differentiation medium containing 50 µg/mL ascorbic acid 2‐phosphate, 7–10 nM dexamethasone and 50 µg/mL indomethacin (all from Sigma). After 21 days of incubation, intracellular lipid accumulation was assessed by Oil Red O staining (Sigma).

#### 2.1.9 Cell Viability

Cell viability was assessed using the MTT assay at 24 and 72 h after seeding MSCs onto chitosan and chitosan‐fennel scaffolds. First, the desired scaffolds were placed into 96‐well plates in triplicate. Next, approximately 5000 ovine fetal BM‐MSCs were seeded into each well. A group without a scaffold served as the negative control. Around 100 µL of supplemented DMEM‐HG medium was added to each well, and the plates were incubated under standard cell culture conditions. After the incubation period, the culture medium was removed from each well. Around 100 µL of MTT solution was then added to each well and incubated for 4 h. Following the removal of the supernatant, 100 µL of DMSO solution was added to each well to solubilize the formazan crystals. The optical density (OD) at 570 nm was measured using an ELISA reader to determine cell viability.

#### 2.1.10 Osteogenic Differentiation Induction

To induce osteogenic differentiation, BM‐MSCs were seeded in 6‐well plates onto chitosan and chitosan/fennel hydrogels (containing 0.5% or 1% fennel extract) or cultured with 0.5% or 1% fennel extract alone. The culture medium was then replaced with an osteogenic differentiation medium consisting of DMEM‐LG supplemented with 10% FBS, 1% antibiotic, β‐glycerophosphate, ascorbic acid 2‐phosphate and dexamethasone. Cells were maintained in this osteogenic differentiation medium for 21 days, with the medium being refreshed every 3 days.

#### 2.1.11 Calcium Content Assay and Alkaline Phosphatase Activity

At the 21st day post‐osteogenic induction, total protein was extracted from differentiated MSCs in all groups. Cells were lysed using 200 µL of RIPA lysis buffer. The lysate was then centrifuged at 15,000 × *g* for 15 min at 4°C to remove cell debris. The supernatants were collected, and ALP activity was determined using an ALP activity assay kit (Pars Azmoon, Iran). The enzyme activity was normalized to total protein content, which was determined using a separate protein assay kit (Pars Azmoon, Iran). Calcium deposition within the hydrogels was quantified using the Cresolphthalein Complex one method which in calcium ions (Ca2+) react with o‐Cresolphthalein Complexone in an alkaline solution to form a violet‐coloured complex. The intensity of this purple colour is directly proportional to the concentration of calcium in the sample. This colour intensity is then measured using a spectrophotometer at a specific wavelength (typically around 570–580 nm). To perform, this study, following removal of the culture medium, the hydrogels were washed with PBS and then incubated with 0.6 N HCl for 4 h at 4°C on a shaker to extract the calcium. The extracted calcium solutions were then analyzed using a commercially available calcium assay kit (Pars Azmoon, Iran). The optical density of the samples was measured at 570 nm using a microplate reader (BioTek Instruments, USA).

#### 2.1.12 Alizarin Red Staining

After 21 days of osteogenic induction, 5 mL of 4% paraformaldehyde was added to each well‐containing cell or cell‐laden scaffold for 10 min at 4°C to fix the cells. Subsequently, the cells were stained with Alizarin Red S (pH 4.2) at 25°C. Images were captured using a digital camera.

#### 2.1.13 Expression of Osteogenic Genes With Real‐Time PCR

Cells grown on hydrogels or in two‐dimensional culture were washed three times with PBS. Gene expression levels of osteogenic markers, including Runx2, ALP and Col‐1A1, were assessed by RT‐PCR after 3 weeks of osteogenic induction. RNA was isolated from BM‐MSCs cultured in 6‐well plates using the TRIzol Plus RNA Purification Kit according to the manufacturer's instructions. RNA purity was assessed by measuring the absorbance at 260 and 280 nm using a spectrophotometer. The PrimeScript RT Reagent Kit for RT‐PCR was used to reverse‐transcribe 500 ng of the isolated RNA into cDNA. Real‐time PCR was performed using a SYBR Green II PCR Kit on a Rotor‐Gene 6000 real‐time PCR cycler. The PCR cycling conditions were 40 cycles of denaturation at 95°C for 10 s and annealing at 60°C for 30 s.

An SYBR Green II PCR Kit was used to run RT‐PCR on a Rotor Gene 6000 real‐time PCR cycler. The PCR cycling conditions were 40 cycles of denaturation at 95°C for 10 s and annealing at 60°C for 30 s. Fluorescence data were acquired at 60°C during the elongation phase of each PCR cycle. The primer sequences for the RT‐PCR are listed in Table 1. Relative gene expression levels were normalized to the expression of the GAPDH gene

**TABLE 1:** The genes that were examined by real‐time PCR, the forward and reverse primers, and annealing temperatures of the products.

Name of primer	Sequence (5′→3′)	Length	Tm
Runx2	For: CCGCCGGACTCGAACTG	17	60
	Rev: GAGAGGCGCAGGTCTTGATG	20	
Col1A	For: CATGACCGAGACGTGTGGAA	20	60
	Rev: CATTCGTCCGTGGGGACTTT	20	
ALP	For: GGTACTTTGGGCGTAACAGCAG	22	60
	Rev: CGGAGAAGCATGAGTCACAGAG	22	
GAPDH	For: ATCGTGGAGGGACTTATGACC	21	60
	Rev: CGCCAGTAGAAGCAGGGATG	20	

#### 2.1.14 Statistical Analysis

Each experiment was performed in triplicate. Data was presented as mean ± standard deviation (SD). One‐way analysis of variance (ANOVA) was used to compare the experimental groups. They were further analyzed with the Duncan test. Statistical significance was set at a p‐value of less than 0.05. All statistical analyses were performed using SPSS software (SPSS Inc., Chicago, IL, USA).

## 3 Results

### 3.1 Results of the GC‐MS Chromatogram Analysis and Chemicals Found in Fennel Hydroalcoholic Extract

**FIGURE 1:**
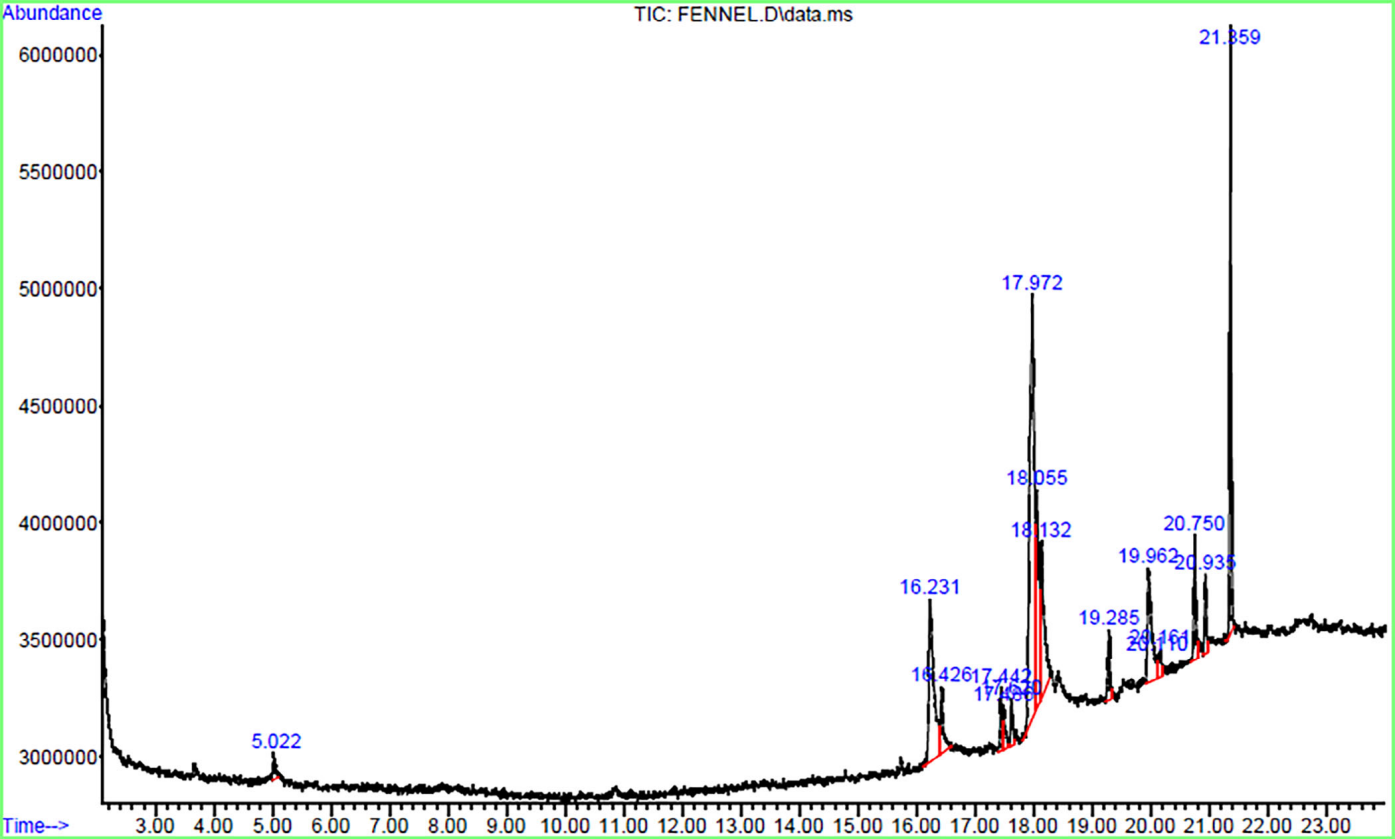
GC‐MS chromatogram of hydroalcoholic extract of *Foeniculum vulgare* seed. Sixteen peaks were observed in the chromatogram, of which 12 were identified as major components in fennel hydroalcoholic extract.

Table 2 presents the results of GC‐MS analysis of the fennel hydroalcoholic extract. The GC‐MS chromatogram of the 16 peaks of the compounds detected is shown in Figure 1. Chromatogram GC‐MS analysis of the extract of *F. vulgare* showed the presence of twelve major peaks and the components corresponding to the peaks were determined as follows: The first set‐up peak was determined to be n Hexadecanoic acid. The second peak is indicated to be carbon monoxide. The next peaks considered to be Nitrogen, Nitrogen, 9,12‐Octadecadienoic acid (Z,Z), 9,12‐Octadecadienoic acid (Z,Z), 9‐Eicosenoic acid, (Z)‐, Hexanoic acid, 2‐ethyl‐, oxybis(2,1‐ethanediyloxy‐2,1‐ethanediyl) ester, Hexadecenoic acid, Z‐11‐, Bis(2‐ethylhexyl) phthalate.

**TABLE 2:** FT‐IR peak values of *Foeniculum vulgare*.

No.	RT	Compound	Chemical formula	Molecular weight	Peak percentage
1	16.23	n Hexadecanoic acid	C16H32O2	256	3/12
2	16.42	Carbon monoxide	CO	28	92/2
3	17.48	Nitrogen	N2	28	59/1
4	17.60	Nitrogen	N2	28	39/1
5	17.97	9,12‐Octadecadienoic acid (Z,Z	C18H32O2	280	41/30
6	18.05	9,12‐Octadecadienoic acid (Z,Z	C18H32O2	280	12/9
7	19.2	9,12‐Octadecadienoic acid (Z,Z	C20H38O2	280	71/8
8	19.28	9‐Eicosenoic acid, (Z)‐	C30H32N2O2	310	20/2
9	19.96	9‐Octadecenamide, (Z)‐	C18H35NO	281	09/7
10	20.75	Hexanoic acid, 2‐ethyl‐, oxybis(2,1‐ethanediyloxy‐2,1‐ethanediyl) ester	C24H46O7	446	82/3
11	20.93	Hexadecenoic acid, Z‐11‐	C16H30O2	254	20/2
12	21.35	Bis(2‐ethylhexyl) phthalate	C24H38O4	390	06/14

### 3.2 Findings From Total Phenolic and Flavonoid Compounds Fennel Hydroalcoholic Extract

The quantities of total phenolic components (standardized based on gallic acid equivalents) and total flavonoid compounds (standardized based on quercetin equivalents) per µg/mL of extract Table 3 indicate that the hydroalcoholic extract of fennel contained 17.3 µg/µL of flavonoid components and 12.6 µg/µL of total phenols.

**TABLE 3:** Total phenolic and flavonoid contents in the hydroalcoholic extract of fennel.

Sample	Total phenolic content standardized against gallic acid (µg/mL)	Total flavonoid content standardized against quercetin (µg/mL)
Fennel seed hydroalcoholic extract	12.6 ± 0.35	17.3 ± 0.22

#### 3.2.1 Scaffold Characterization

**FIGURE 2:**
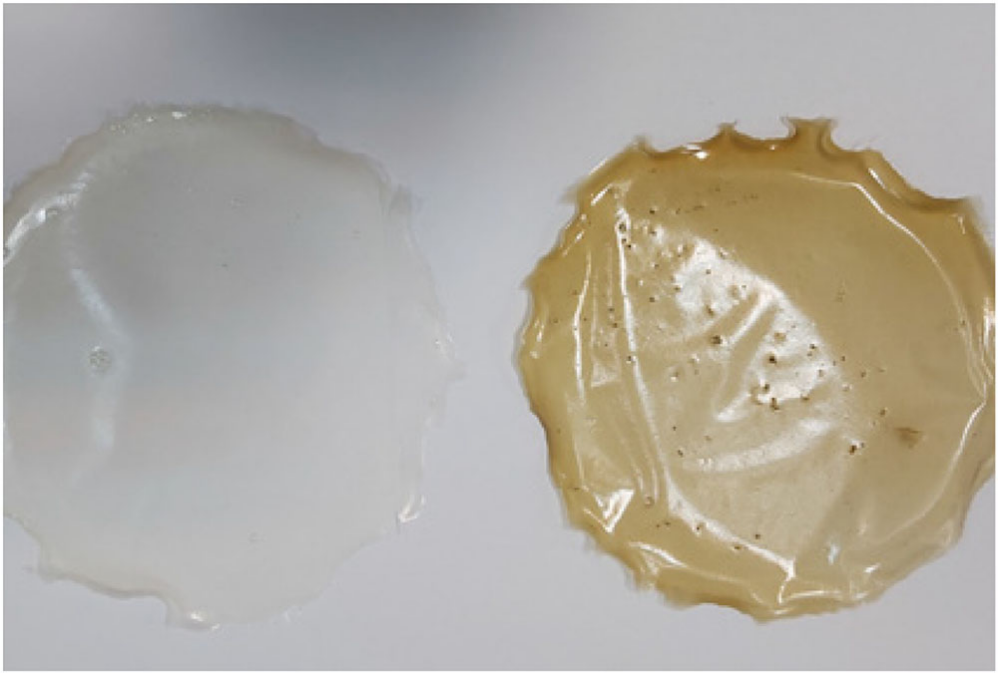
Shows a chitosan film with fennel hydroalcoholic extract (1%) on the right side and a film without extract on the left.

**FIGURE 3:**
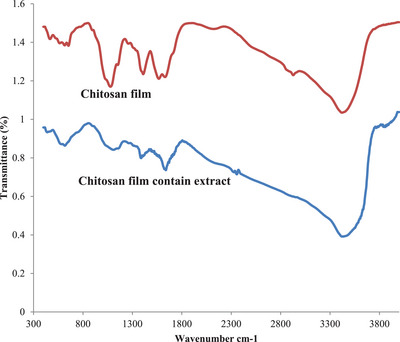
Shows the FT‐IR spectrum of chitosan film without extract, and chitosan film containing hydroalcoholic fennel extract (1%).

Figure 2 shows chitosan and chitosan‐extract‐containing hydrogel films. FTIR spectroscopy was employed to characterize the functional groups within the chitosan film and the chitosan film containing the hydroalcoholic fennel extract. The spectra were recorded over the range of 4000–500 cm^−1^. FTIR analysis was performed to investigate potential intermolecular interactions between the components of the scaffold. A) The chitosan film exhibited characteristic peaks in the FTIR spectrum. A broad peak at 3420 cm^−1^ corresponds to the stretching vibrations of ─OH and ─NH2 groups. The peak at 2927 cm^−1^ is attributed to the stretching vibrations of ─CH groups. Other prominent peaks include the C─O─C stretching vibration at 1049 cm^−1^ and the amide II band at 1567 cm^−1^, characteristic of the ─NH group in chitosan (Figure 3).

**FIGURE 4:**
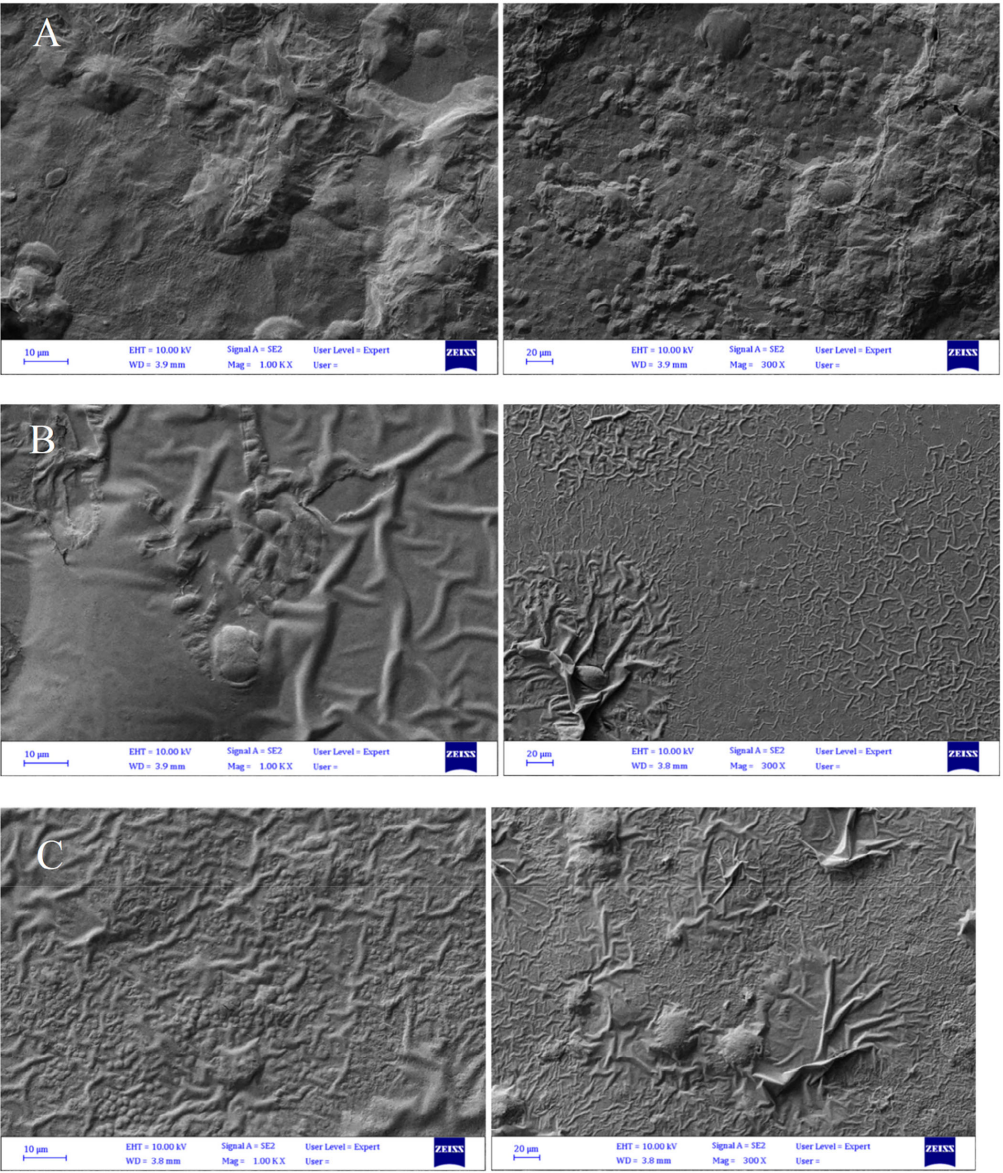
Shows SEM images of three different chitosan films: (A) without extract, (B) with 0.5% fennel hydroalcoholic extract, and (C) with 1% extract.

SEM was used to reveal the surface morphology of chitosan films without extract and containing 0.5% and 1% fennel hydroalcoholic extract. As seen in the figures (Figure 4a,b,c), the surface morphology of the chitosan film without extract exhibited protrusions on the surface and appeared heterogeneous.

#### 3.2.2 Morphology of Ovine Fetal BM‐MSCs

**FIGURE 5:**
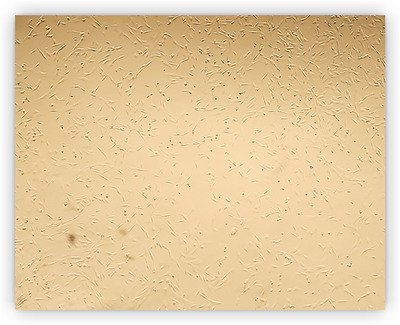
Morphology of Ovine fetal bone marrow‐derived mesenchymal stem cells (P3).

MSCs were isolated from ovine fetal bone marrow and cultured in vitro. After 48 h of initial culture, non‐adherent cells were removed, and adherent fibroblast‐like cells were observed to attach to the bottom of the culture flask. These cells exhibited rapid proliferation during the initial culture period. They typically displayed spindle‐ or triangular morphologies, possessing a large, oval nucleus. The cell colonies initially expanded outward before undergoing a characteristic contraction phase. Confluence was typically reached within 5 days (Figure 5).

#### 3.2.3 Adipose and Osteoblast Cell Differentiation in BM‐MSCs

**FIGURE 6:**
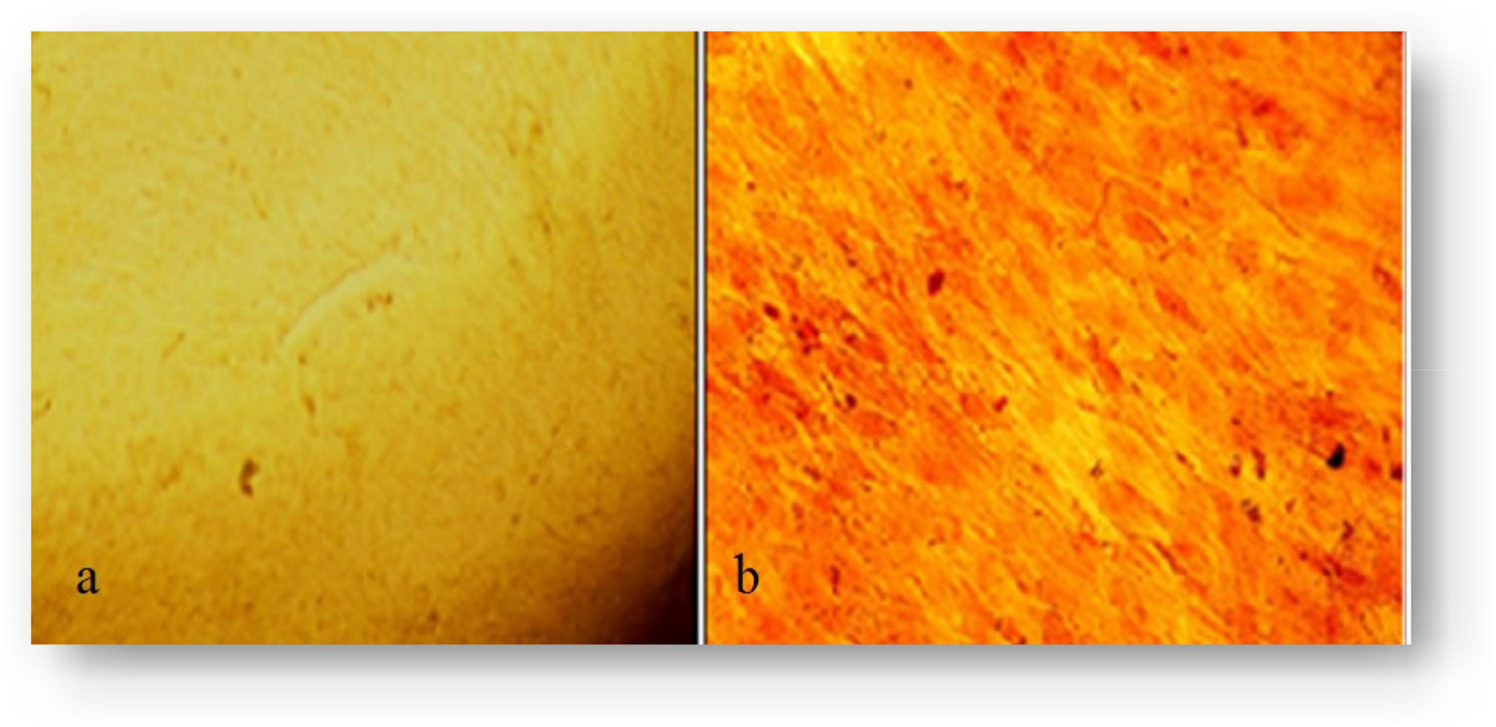
Alizarin red S staining confirming the differentiation of Ovine fetal bone marrow‐derived mesenchymal stem cells into the osteoblast at day 21 (a), control and (b), osteogenic media.

**FIGURE 7:**
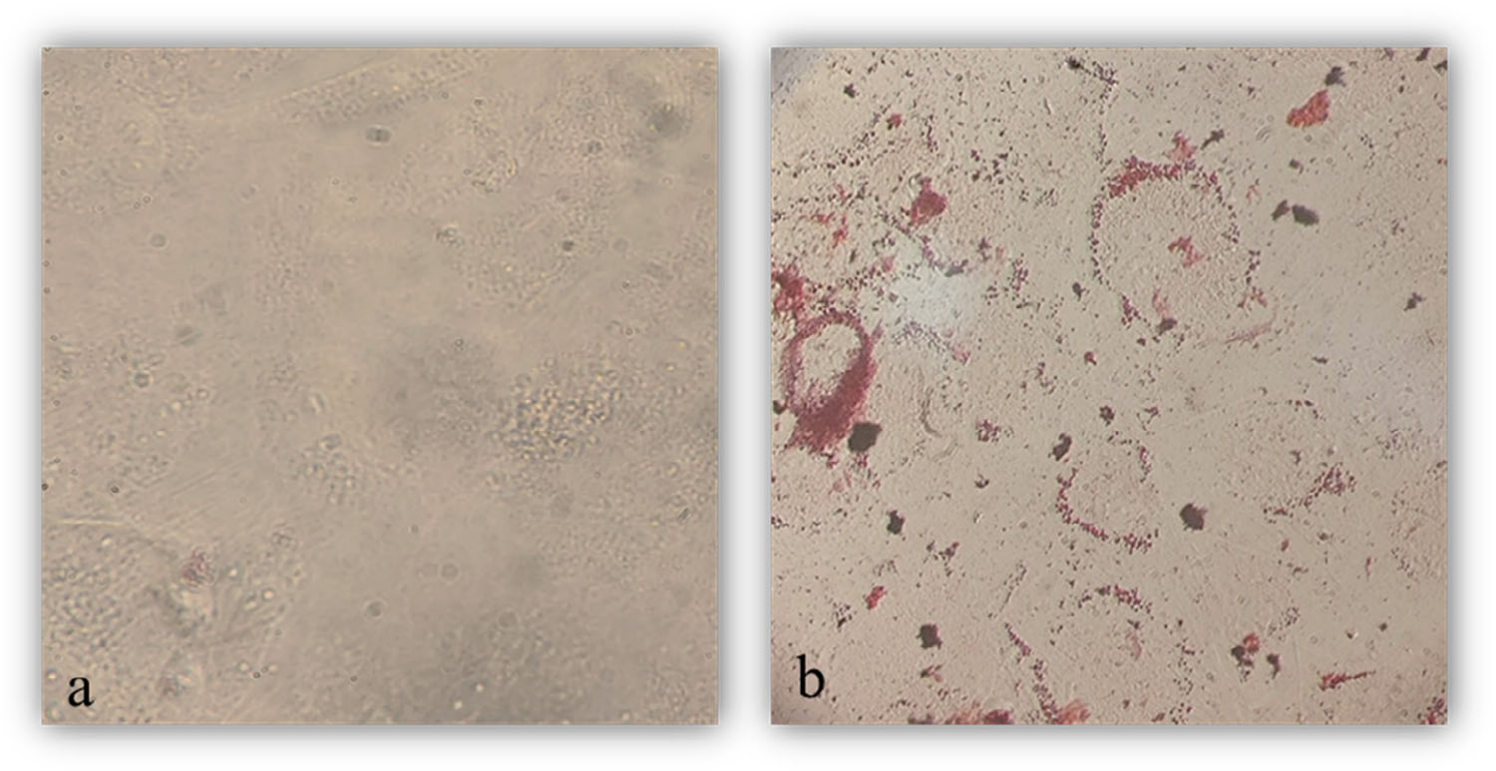
Oil red O staining confirming the differentiation of Ovine fetal bone marrow‐derived mesenchymal stem cells into the adipose cell at day 21 (a), control and (b), adipogenic media.

After 21 days of incubation in osteogenic differentiation media, Alizarin Red S staining was performed to assess the formation of mineralized nodules, indicating osteoblast differentiation (Figure 6). Similarly, adipogenic differentiation was confirmed by Oil Red O staining, which visualized the accumulation of intracellular lipid droplets within the cells (Figure 7).

### 3.3 Apoptosis Assay

**FIGURE 8:**
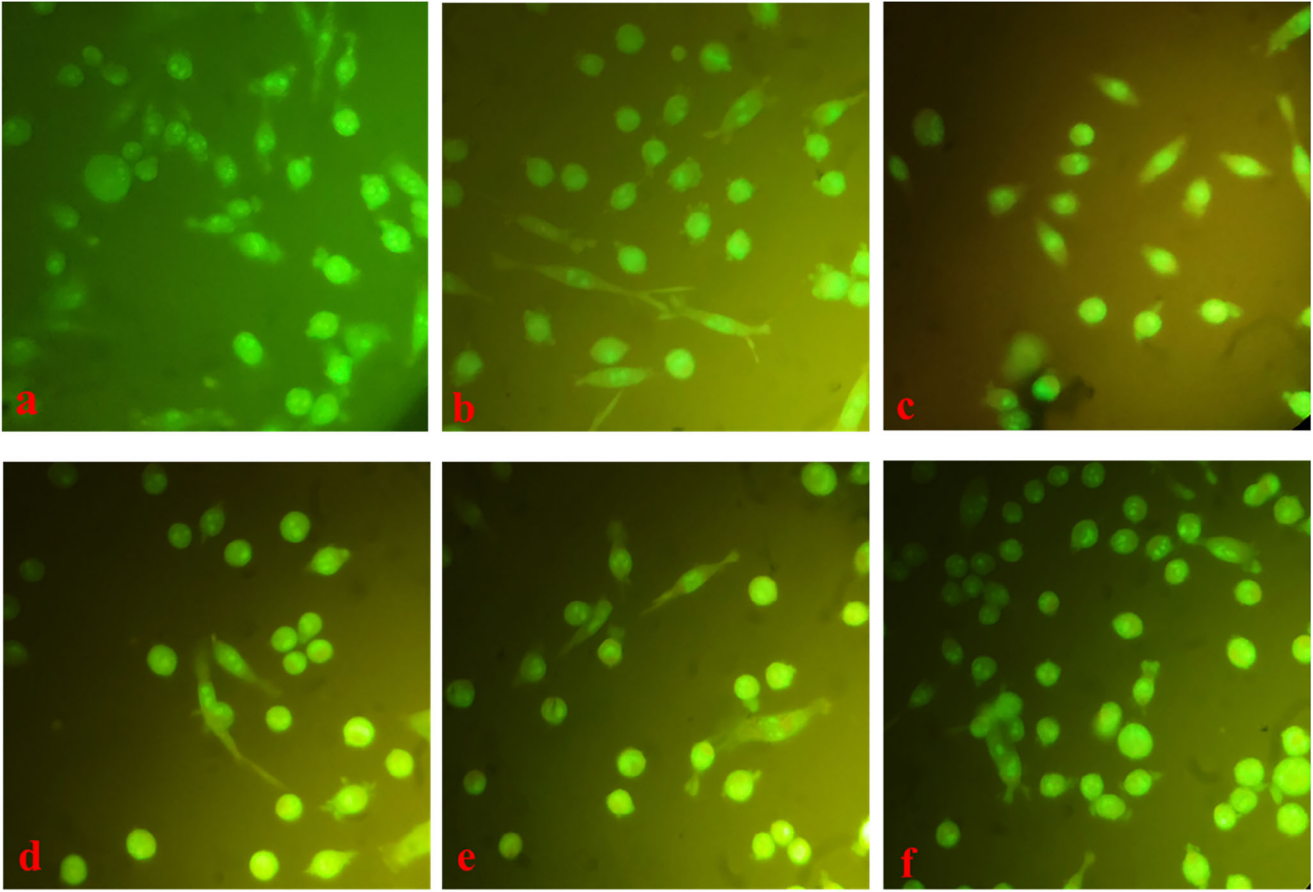
Shows the simultaneous staining of fibroblastic cells with ethidium bromide and acridine orange (a) control, (b) 0.5% of fennel extract, (c) 1% of fennel extract, (d) chitosan film groups, (e) chitosan film containing 0.5% fennel extract; (f) chitosan film containing 1% fennel extract after 72 h.

As illustrated in the images, none of the treatment groups used in this study induced apoptosis in the cultured fibroblastic cells after 72 h. Staining with ethidium bromide and acridine orange revealed no evidence of cell death in any of the treatment groups (Figure 8).

### 3.4 Cell Viability

**FIGURE 9:**
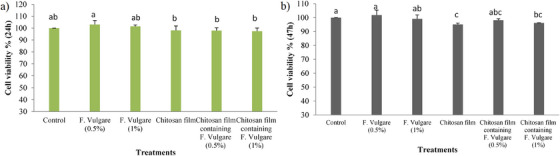
Analysis of the viability rate of foetal Ovine BM‐MSCs at 24(a) and 72(b) hours. (3 replicates; mean ± SD; *p* <0.05).

The MTT assay revealed that the 0.5% fennel extract group exhibited the highest rate of cell proliferation after 24 h compared to the chitosan film, the chitosan film containing 0.5% extract and the chitosan film containing 1% extract. However, this difference was not statistically significant compared to the control and the 1% fennel extract groups (Figure 9a; *p* > 0.05). The results of the MTT assay demonstrated that the 0.5% extract treatment group significantly enhanced the proliferation of BM‐MSCs compared to the chitosan film group and the chitosan films containing 0.5% and 1% fennel hydroalcoholic extract after 72 h (*p* < 0.05). However, no statistically significant difference in cell proliferation was observed between the 0.5% extract treatment group and the control or the 1% extract treatment groups (*p* > 0.05) (Figure 9b).

### 3.5 Hydroxyapatite Sediment Quantification Following Alizarin Red Staining

**FIGURE 10:**
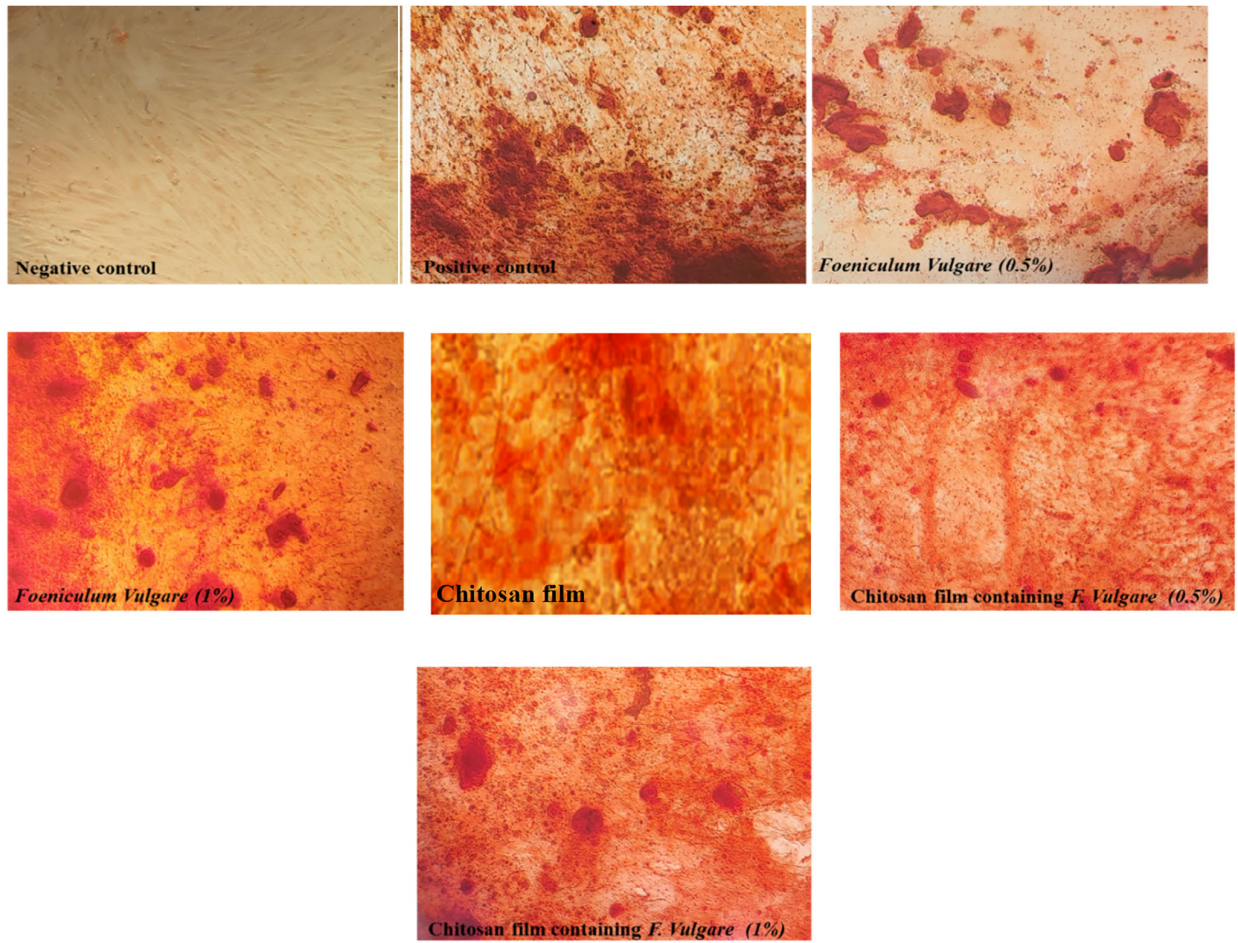
Mineral accumulation in ovine fetal BM‐MSCs after Alizarin red labelling on Day 21 following stimulation of osteocyte differentiation under a microscope.

**FIGURE 11:**
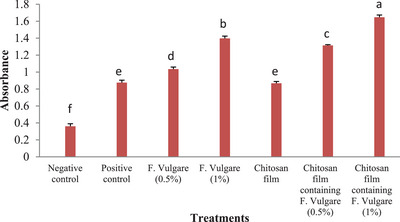
Quantification of the optical density of 10% acetic acid‐dissolved alizarin red staining at a 405 nm wavelength. (Mean ± SD; *p* < 0.05; *n* = 3 biological samples).

Alizarin Red S staining revealed the presence of mineralized nodules, visualized as red‐stained areas, in the osteogenic differentiated BM‐MSC cultures after 21 days. These mineralized deposits were observed in the treatment groups containing chitosan films with extract and in the groups treated with extract alone but were absent in the control group (Figure 10). Following alizarin red staining, the quantification of calcium deposits within the hydrogels is presented in Figure 10. A significant increase in calcium deposition was observed in the group cultured on the chitosan film containing 1% fennel extract compared to the control group and all other treatment groups (*p* < 0.05). Furthermore, the chitosan film containing 1% fennel extract also significantly enhanced calcium deposition compared to the chitosan‐only group, the 0.5% fennel extract group, and the group cultured with 0.5% fennel extract alone as illustrated in Figure 11, (*p* < 0.05). These findings indicate that the chitosan film incorporating 1% fennel extract significantly promoted osteoblast differentiation and mineralization compared to the other groups.

### 3.6 Ovine Foetal BM‐MSCs Mineralization

**FIGURE 12:**
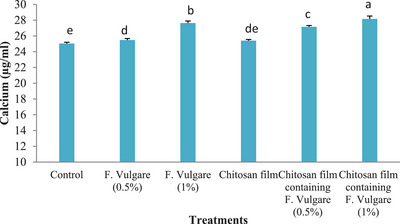
Ovine fetal BM‐MSC mineralization 21 days following osteocyte differentiation stimulation. (Mean ± SD; *p* < 0.05; *n* = 3 biological samples).

Figure 12 illustrates the mineralization rate of ovine foetal BM‐MSCs after 21 days of osteogenic differentiation. The results demonstrate that the chitosan film containing 1% fennel extract significantly enhanced mineralization activity compared to the control and other treatment groups (*p* < 0.05). It also depicts the mineralization rate of ovine fetal BM‐MSCs after 21 days of osteogenic differentiation. Compared to the control group, the chitosan film group, the 0.5% fennel extract group and the chitosan film containing 0.5% fennel extract, the group cultured on the chitosan film with 1% fennel extract exhibited significantly higher mineralization activity (Figure 12), (*p* < 0.05)

#### 3.6.1 Alkaline Phosphatase (ALP) Activity

**FIGURE 13:**
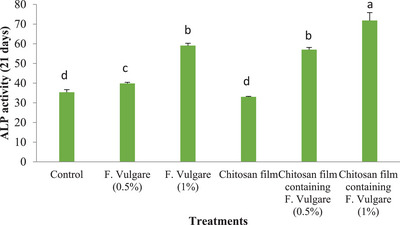
Alkaline phosphatase activity of ovine fetal BM‐MSCs 21 days after osteocyte development (*n* = 4 biological samples; mean ± SD; *p* < 0.05).

Figure 13 illustrates ALP activity in ovine foetal BM‐MSCs after 21 days of osteogenic differentiation. A significant increase in ALP activity was observed in cells cultured on the chitosan film containing 1% fennel extract compared to the control group, the chitosan film group, the 0.5% fennel extract group, and the chitosan film containing 0.5% fennel extract (*p* < 0.05). Similarly, ALP activity was significantly higher in cells cultured with 1% fennel extract compared to the control group, the chitosan film group, the 0.5% fennel extract group and the chitosan film containing 0.5% fennel extract (*p* < 0.05)

### 3.7 Osteogenic Genes Expression

**FIGURE 14:**
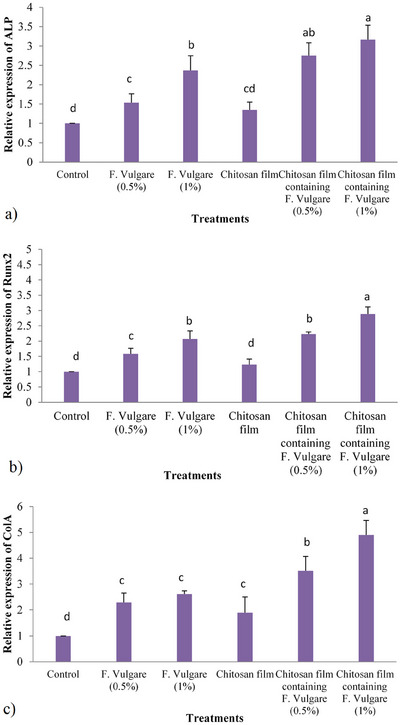
Expression analysis of the osteogenic‐specific genes in the experimental groups. Statistical significance was defined at *p* < 0.05 applying analysis of variance (ANOVA) using SPSS software.

Real‐time PCR data for the expression of osteogenic genes are presented in Figures (14a,b,c).

#### 3.7.1 ALP Gene Expression

Compared to the control group, all treatment groups except for the chitosan film containing 1% fennel extract significantly downregulated ALP gene expression in ovine fetal BM‐MSCs (*p* < 0.05). No significant difference in ALP gene expression was observed between the groups treated with chitosan films containing 0.5% and 1% fennel extract (*p* > 0.05).

#### 3.7.2 Runx2 Gene Expression

The chitosan film containing 1% fennel extract significantly upregulated Runx2 gene expression compared to all other treatment groups (*p* < 0.05). No significant difference in Runx2 gene expression was observed between the groups treated with chitosan films containing 0.5% and 1% fennel extract (*p* > 0.05).

#### 3.7.3 ColA1 Gene Expression

The chitosan film containing 1% fennel extract significantly upregulated ColA1 gene expression compared to all other treatment groups (*p* < 0.05). Furthermore, the chitosan film containing 0.5% fennel extract also significantly increased ColA1 gene expression compared to the control group, the chitosan film group and the group treated with 0.5% fennel extract alone (*p* < 0.05).

## 4 Discussion

This study investigated the osteogenic differentiation potential of ovine foetal BM‐MSCs seeded on chitosan hydrogel scaffolds incorporating fennel seed extract. Chitosan scaffolds were prepared and characterized. Fibroblast‐like adherent cells from the third passage were used for subsequent experiments. MTT assay demonstrated increased cell proliferation at 24 and 72 h in all groups. Acridine Orange/Ethidium Bromide (AO/EB) staining confirmed the absence of cytotoxicity in all treatment groups. To assess osteogenic differentiation, the expression of osteoblastic genes (RUNX2, COL‐1A1, and ALP), ALP activity and calcium deposition were evaluated after 21 days. Gene expression analysis revealed that the chitosan film containing 1% fennel extract significantly upregulated the expression of Runx2, Col‐1A1 and ALP genes compared to all other treatment groups. The results demonstrated that the chitosan scaffold containing 1% fennel extract significantly enhanced osteogenic differentiation, as evidenced by increased ALP activity, calcium deposition and expression of osteoblastic genes.

To the best of our knowledge, this study is the first to investigate the effect of fennel seed extract on the osteogenic differentiation potential of ovine foetal bone BM‐MSCs seeded on a chitosan hydrogel scaffold.

Suitable cells for tissue engineering possess several key characteristics, including ease of culture and the ability to interact effectively with the surrounding environment (Wolff et al. 2011). In bone tissue engineering, stem cells, such as mesenchymal stem cells (MSCs), or pre‐osteoblastic cells are utilized to regenerate bone tissue through cell‐based therapies. To effectively guide these cells towards bone formation, it is crucial to provide them with a culture environment that closely mimics the physiological conditions found within the human body. Today, 3D culture environments have demonstrated superior performance compared to traditional 2D culture systems. Hydrogels are among the most promising scaffolds for tissue engineering due to their excellent biocompatibility, high porosity and remarkable similarity to the extracellular matrix. Furthermore, their soft consistency minimizes the risk of eliciting an inflammatory response from surrounding cells and tissues (Buwalda et al. 2017). Previous studies have reported that MSCs seeded on fibrin hydrogels can differentiate into osteogenic cells (Catelas et al. 2006). Naito and colleagues demonstrated that MSCs seeded on collagen hydrogel scaffolds exhibited enhanced osteogenic differentiation compared to 2D culture conditions (Naito et al. 2011). The unique structural and multidimensional properties of chitosan make it an attractive biomaterial for bone tissue engineering applications (Pillai et al. 2009). In one study, gelatin‐chitosan 3D hybrid hydrogels, supplemented with human platelet lysate, demonstrated the ability to support human mesenchymal stem cell (hMSC) proliferation and promote osteogenic differentiation. These hydrogels exhibited excellent biocompatibility and biodegradability, with favourable chemical, morphological and mechanical properties that collectively support cell growth, bone differentiation and mineralization. These findings suggest that gelatin‐chitosan hydrogels have the potential to serve as effective scaffolds for bone tissue regeneration (Re et al. 2019). Chitosan nanofibres have been shown to stimulate osteoblast proliferation and maturation by regulating the expression of Runx2, a key transcription factor involved in osteogenesis (Ho et al. 2014). Chitosan‐collagen composite films significantly upregulated the expression of osteoblast‐specific genes, such as collagen type I (Col1) and Runx2, in MC3T3‐E1 osteoblast‐like cells (Wang et al. 2016). BM‐MSCs were induced to differentiate into osteoblast‐like cells by seeding them into chitosan/polyethylene oxide/nanohydroxyapatite nanofibers (Emamgholi et al. 2019).

Estrogen plays an important role in bone regeneration, and postmenopausal decline in serum estrogen levels is associated with osteoporosis in older women (Nakamura et al. 2007). Estrogen effects on osteoblasts and osteoclasts are through binding to the intracellular estrogen receptor and regulating the production of target proteins (Khalid and Krum 2016). Phytoestrogens with a structure similar to 17 beta‐estradiol (E2) have a protective role against osteoporosis in postmenopausal women (Limer and Speirs. 2004). Fennel seed has phytoestrogenic molecules (Patil et al. 2022). Our findings demonstrate that fennel extract, in combination with a chitosan scaffold, significantly enhanced osteogenic differentiation of MSCs. The chitosan scaffold incorporating 1% fennel seed extract exhibited the highest levels of osteogenic parameters. These results are consistent with previous findings by Mahmoudi et al., who reported that fennel seed extract can promote the proliferation and osteogenic differentiation of hMSCs while demonstrating no cytotoxic effects (Mahmoudi et al. 2013; Suh et al. 2007). In another study, silymarin, as a phytoestrogen was found to induce ovine foetal BM‐MSCs to differentiate into osteoblast‐like cells. Importantly, the study also reported that silymarin did not exhibit any toxicity to the cells. This finding suggests that silymarin may have the potential as a natural compound for promoting bone formation (Morovati et al. 2022). Genistein, a prevalent isoflavone found in soybeans (Glycine max), is classified as a phytoestrogen due to its structural resemblance to human estrogen. This similarity allows it to interact with estrogen receptors α and β (ERα and ERβ), thereby mimicking estrogen's effects. These ER‐mediated actions include promoting bone growth and inhibiting the development of fat tissue (Jaiswal et al. 2019). **Purarin**, another phytoestrogen, has been shown to exhibit **osteoinductive potential in vitro** (Yang et al. 2022). It seems that osteoinductive factors’ effects increase when cells are seeded on the scaffolds. Human endometrial stem cells were cultured on a collagen‐hydroxyapatite scaffold containing a small molecule of pormorphamine. Culturing cells in a 3D environment, especially with the addition of pormorphamine, significantly enhanced their differentiation into bone‐forming osteogenic cells. This was shown by the elevated expression of Col‐1 and RUNX‐2 genes—indicators of bone development—in both the standard 3D scaffold and the pormorphamine‐treated 3D scaffold compared to cells grown in a traditional 2D setting (Bahrami et al. 2017).

A 3D hydrogel composed of fluorenyl‐9‐methoxycarbonyl diphenylalanine (Fmoc‐FF) peptides notably enhanced the transformation of MSCs into bone cells in research conducted by Fouladgar and colleagues. Their findings indicate that these peptides provide attachment points for integrins, thereby activating an intracellular signalling cascade vital for this differentiation process (Fouladgar et al. 2025).

## 5 Conclusion

This study demonstrated that chitosan hydrogels incorporating fennel seed extract can effectively promote the osteogenic differentiation of ovine fetal BM‐MSCs. These findings highlight the potential of fennel seed extract as a natural bioactive compound for enhancing bone tissue engineering strategies. The 3D chitosan scaffold provided a suitable environment for cell growth and differentiation, further supporting the observed osteogenic effects. This study contributes to the growing body of evidence supporting the use of natural compounds and biocompatible scaffolds for bone tissue regeneration.

## Author Contributions

**Kosar Heidari**: funding acquisition. **Tayebeh Mohammadi**: investigation, funding acquisition, writing – original draft, writing – review and editing, methodology, formal analysis. **Leila Soltani**: investigation, writing – original draft, methodology, formal analysis. **Mehrdad PooyanMehr**: funding acquisition.

## Ethics Statement

Animal husbandry and handling were conducted in accordance with the guidelines of Animal Ethics Committee (Permission number: IR.RAZI.REC.1401.052) of Razi University, Kermanshah, Iran.

## Conflicts of Interest

The authors declare no conflicts of interest.

## Data Availability

The data that support the findings of this study are available from the corresponding upon reasonable request.
